# Multi-Gas Detection System Based on Non-Dispersive Infrared (NDIR) Spectral Technology

**DOI:** 10.3390/s22030836

**Published:** 2022-01-22

**Authors:** Manlin Xu, Bo Peng, Xiangyi Zhu, Yongcai Guo

**Affiliations:** 1 Key Laboratory of Optoelectronic Technology and Systems of Ministry of Education, College of Optoelectronic Engineering, Chongqing University, Chongqing 400044, China; manlinxu@cqu.edu.cn (M.X.); XiangyiZhu@cqu.edu.cn (X.Z.); 2 Liangjiang School of Artificial Intelligence, Chongqing University of Technology, Chongqing 401135, China; pengbo86@126.com

**Keywords:** non-dispersive infrared, multi-gas detection, gas sensor

## Abstract

Automobile exhaust gases, such as carbon dioxide (CO_2_), carbon monoxide (CO), and propane (C_3_H_8_), cause the greenhouse effect, photochemical smog, and haze, threatening the urban atmosphere and human health. In this study, a non-dispersive infrared (NDIR) multi-gas detection system consisting of a single broadband light source, gas cell, and four-channel pyroelectric detector was developed. The system can be used to economically detect gas concentration in the range of 0–5000 ppm for C_3_H_8_, 0–14% for CO, and 0–20% for CO_2_. According to the experimental data, the concentration inversion model was established using the least squares between the voltage ratio and the concentration. Additionally, the interference coefficient between different gases was tested. Therefore, the interference models between the three gases were established by the least square method. The concentration inversion model was experimentally verified, and it was observed that the full-scale error of the sensor changed less than 3.5%, the detection repeatability error was lower than 4.5%, and the detection stability was less than 2.7%. Therefore, the detection system is economical and energy efficient and it is a promising method for the analysis of automobile exhaust gases.

## 1. Introduction

Previous studies have demonstrated that the pollution caused by automobile exhaust gases accounts for 60% of the total urban air [1]. Automobile exhaust has a complex composition; its main components are CO, CO_2_, hydrocarbons, and suspended particles. These emissions have severely polluted the environment and endangered human health [2,3]. Therefore, a multi-gas concentration (C_3_H_8_, CO, and CO_2_) detection system is required to assess automobile exhaust emission levels. It has great significance for the protection of urban air quality.

Multiple researchers have conducted studies on multi-gas detection systems [4]. Besson reported a multi-gas photoacoustic sensor (CH_4_, H_2_O, and HCl) based on tunable diode laser absorption spectroscopy (TDLAS) in the near-infrared region [5]. TDLAS has the advantages of high precision, and high sensitivity, but it also has disadvantages; for example, it is single wavelength, and can only be used for one type of gas [6,7]. Betty reported a multi-gas sensor (NH_3_, H_2_S, and NO_2_) using a SnO_2_ nanocrystalline thin film [8]. Although metal oxide sensors are economical and have a quick response in gas sensing applications, they have poor stability, are prone to humidity interference, and cannot be operated at high temperatures [6,7,8,9,10,11]. An ultra-sensitive and selective quartz-enhanced photoacoustic spectroscopy (QEPAS) sensor platform was studied for the detection of carbon monoxide (CO) and nitrous oxide (N_2_O) [12]. NO and CO in the Shanghai urban atmosphere were measured using a portable optical sensing instrument based on room-temperature pulsed QC lasers during the EXPO 2010 [13]. Qiao reported on an ultra-highly sensitive light-induced thermoelastic spectroscopy (LITES) based carbon monoxide (CO) sensor exploiting custom quartz tuning forks (QTFs) as a photodetector, a multi-pass cell and a mid-infrared quantum cascade laser (QCL) for the first time [14].

Non-dispersive infrared (NDIR) detectors have been extensively used for multi-gas monitoring [13,14,15]. Compared with other methods, the NDIR technique is considered the simplest approach due to its moderate sensitivity and fast response [16,17,18]. Furthermore, NDIR detectors require low maintenance and are more economical than other gas detection systems. These detectors have been used to measure the concentration of more than 100 types of gases. Wang studied a multi-gas sensor using the galvanometer modulation on NDIR and detected gas concentrations in the range of 0–10% for CO and CO_2_ [19]. Xie proposed a weak signal-processing circuit to measure CO, CO_2_, and C_3_H_8_ using a NDIR detector [20]. Tan developed a NDIR-based three-gas detection system that could detect gas concentration in the range of 0–5% for methane (CH_4_), 0–4.45% for CO, and 0–4.8% for CO_2_ [21]. Liu reported on a NDIR detector for the detection of automobile exhausts (mainly CO and CO_2_) [22]. Dong proposed a multi-gas sensor system for the detection of gas concentration in the range from 0 to 0.25% for CO, CO_2_, and CH_4_, using the time division multiplexing (TDM) technique [23]. Villar developed a space sensor that measured CO and CO_2_ concentration using NDIR. The hardware was designed with a rugged and viable technology for multiple sensor applications in a variety of environments [24]. Liu developed a highly compact NDIR sensor capable of gas-mixture detection (CO, CO_2_, CH_4_, H_2_CO, NH_3_, and NO) with a volume fraction in the range from 0 to 4% [25].

Although the abovementioned methods are capable of gas mixture detection, they have not been applied to the detection of gas mixtures at a higher concentration, such as automobile exhaust gases. In this study, automobile exhaust gases, such as C_3_H_8_, CO, and CO_2_, were detected. A single broadband light source (IRL715, Perkin Elmer) and four-channel pyroelectric detector (LRM-284, InfraTec, Germany) were used to ensure multi-gas detection, and that the gas concentration was in the range of 0–5000 ppm for C_3_H_8_, 0–14% for CO, and 0–20% for CO_2_. A common gas cell fabricated from gold-coated stainless steel cylinders with an inner radius of 4 mm and an optical path length of 108 mm was used in the NDIR detector. A filter circuit was designed for the weak signal from the detector, and a preamplifier was employed to enhance the signal, which was acquired by an analog to digital converter (AD7606, Analog Devices, USA). The signal was processed according to the infrared optical dual-wavelength detection technology. The concentration inversion model was developed using the least squares between the voltage ratio and the concentration. Subsequently, the mutual interference model between the three gases was established. It was verified that the concentration inversion and mutual interference models were successfully established. Furthermore, the analysis showed that the NDIR detector had an appropriate full-scale error, repeatability, and stability. The results of this study will provide an effective reference for automobile exhaust detection.

## 2. Theory

### 2.1. Non-Dispersive Infrared

According to the Beer–Lambert law, gas concentration can be derived by Equation (1):

(1) $$I\left(\lambda \right)=I_{0}\left(\lambda \right)e^{-kcL}$$

where λ is the wavelength of the incident light (nm), c is concentration (ppm), k represents the correlation coefficient of gas absorption, which is related to the absorption property of the target gas and the selected filter, and L represents the optical path length (mm). I_0_(λ) and I(λ) are the light intensity before and after absorbing the target gas, respectively.

To ensure an accurate gas concentration measurement, a multi-channel pyroelectric detector and optical filters were used in this detection system. Based on the HITRAN (high-resolution transmission) molecular absorption database [26], it was observed that the absorption bands of CO, CO_2_, and C_3_H_8_ were located in the range of 3–5 μm, and an overlap between these bands was absent. The main infrared absorption peaks of the above three gases do not overlap. Filters are used to avoid cross interference caused by overlapping edge absorption peaks. A suitable filter is one which can only pass wavelengths that a gas can absorb. The characteristic parameters of the four-channel optical filter are shown in Table 1.

### 2.2. Algorithm Design

The infrared light source emitted a continuous spectrum to the gas cell, and the multi-channel detector selectively received the infrared light corresponding to the optical filter. I_ref_ and I_gas_ are the infrared light intensity before and after absorbing the target gas, respectively. The output voltages of the detection channel, U_gas_ (mV), and reference channel, U_ref_ (mV), after the output signal of the detector was filtered and amplified are given by Equations (2) and (3), respectively.

(2) $$U_{\mathrm{gas}}{=I}_{\mathrm{gas}}R_{\mathrm{gas}}C_{\mathrm{gas}}e^{-\mathrm{kcL}}$$

(3) $$U_{\mathrm{ref}}{=I}_{\mathrm{ref}}R_{\mathrm{ref}}C_{\mathrm{ref}}$$

where $R_{\mathrm{ref}}$ and R_gas_ are system-related constants; C_gas_ and C_ref_ are response factors of the pyroelectric detector. In the specific infrared gas detection system, the above coefficients are constants. To eliminate the interference of factors, such as the instability of the light source power, the required voltage ratio coefficient can be obtained through dividing Equation (2) by Equation (3). Therefore, the relationship between the gas concentration and output voltage can be obtained through Equation (4):

(4) $$\Delta U_{\mathrm{gas}}=\frac{U_{\mathrm{gas}}}{U_{\mathrm{ref}}}=\frac{I\left(\lambda \right)}{I_{0}\left(\lambda \right)}$$

where the parameter ΔU_gas_ is the voltage ratio coefficient, which is used to indicate the relative voltage change with a change in the gas concentration. According to the above three formulas, the parameter ΔU_gas_ can be obtained as follows:

(5) $$\Delta U_{\mathrm{gas}}=\frac{U_{\mathrm{gas}}}{U_{\mathrm{ref}}}=\frac{I_{\mathrm{gas}}R_{\mathrm{gas}}C_{\mathrm{gas}}e^{-\mathrm{kcL}}}{I_{\mathrm{ref}}R_{\mathrm{ref}}C_{\mathrm{ref}}}=\frac{I_{\mathrm{gas}}R_{\mathrm{gas}}C_{\mathrm{gas}}}{I_{\mathrm{ref}}R_{\mathrm{ref}}C_{\mathrm{ref}}}e^{-\mathrm{kcL}}$$

Let $K_{0}=\frac{I_{\mathrm{gas}}R_{\mathrm{gas}}C_{\mathrm{gas}}}{I_{\mathrm{ref}}R_{\mathrm{ref}}C_{\mathrm{ref}}}$, then the ΔU_gas_ can be obtained as follows:

(6) $$\Delta U_{\mathrm{gas}}{=K}_{0}e^{-\mathrm{KCL}}$$

Transforming Equation (6) finally yields the gas concentration in Equation (7):

(7) $$C=-\frac{1}{\mathrm{KL}}\left(\ln\frac{\Delta U_{\mathrm{gas}}}{K_{0}}\right)$$

For the gas detection system, the above coefficients (K, L) are constants. If $\Delta U_{\mathrm{gas}}$ is known, the concentration (C) can be calculated by Equation (8).

(8) $$C=f\left(\Delta U_{\mathrm{gas}}\right)$$

## 3. Experimental System Setup

**Figure 1:**
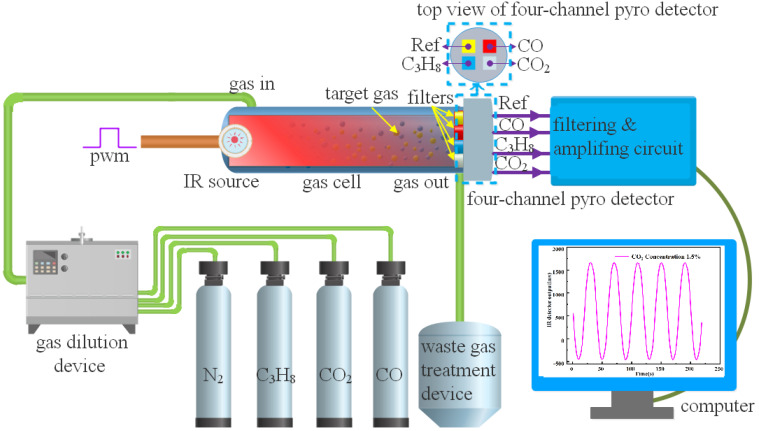
Schematic of the NDIR detector used for multi-gas detection.

Figure 1 shows the experimental setup of the NDIR instrument for multi-gas detection, which consists of an IR source, gas cell, and pyroelectric detector. A stainless gas cell with an optical path length of 108 mm and an inner radius of 4 mm was used. Calcium fluoride plan-convex lenses were used as gas cell windows to enhance the optical power transmission. A four-channel pyroelectric was integrated in the detector to enable simultaneous detection of CO_2_, CO, and C_3_H_8_. The top view of the four-channel detector is shown in the dashed box in Figure 1.

**Figure 2:**
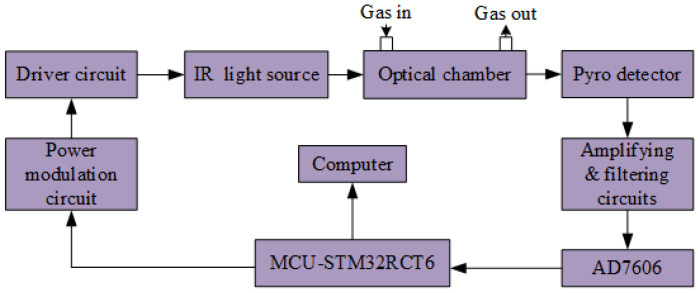
Hardware schematic of the NDIR detector used for multi-gas detection.

**Figure 3:**
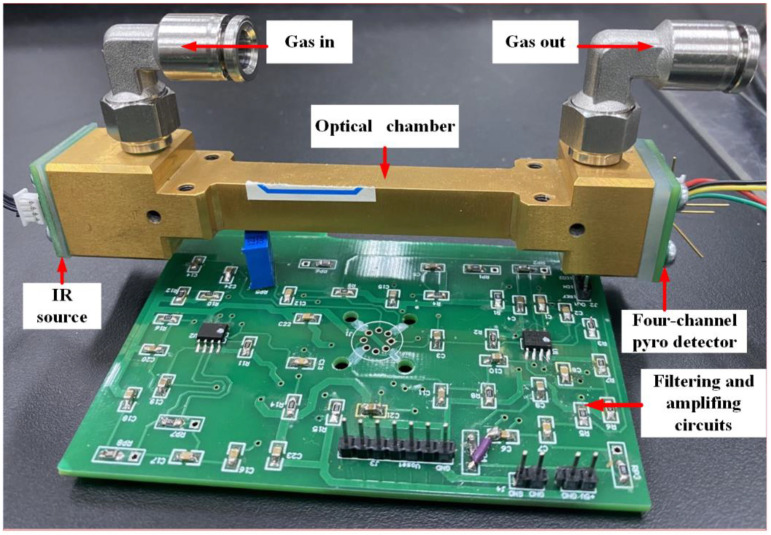
The test system diagram used for multi-gas detection.

Figure 2 shows the hardware schematic of the NDIR instrument for multi-gas detection. In Figure 2, an STM32F103RBT6 (STMicroelectronics, Geneva, Switzerland) was employed as the microcontroller unit (MCU), and it generated a 5-Hz square-wave with 50% duty cycle to drive the infrared (IR) source. As the detector output signal was considerably weak for detection purposes, it was susceptible to external interference. Therefore, a preamplifier was used to enhance the signal acquired by an analog to digital converter (AD7606, ADI, Norwood, MA, USA). Finally, the computer processor was used for signal processing to reduce the noise to a minimum level. The test system diagram used for multi-gas detection is shown in Figure 3.

**Figure 4:**
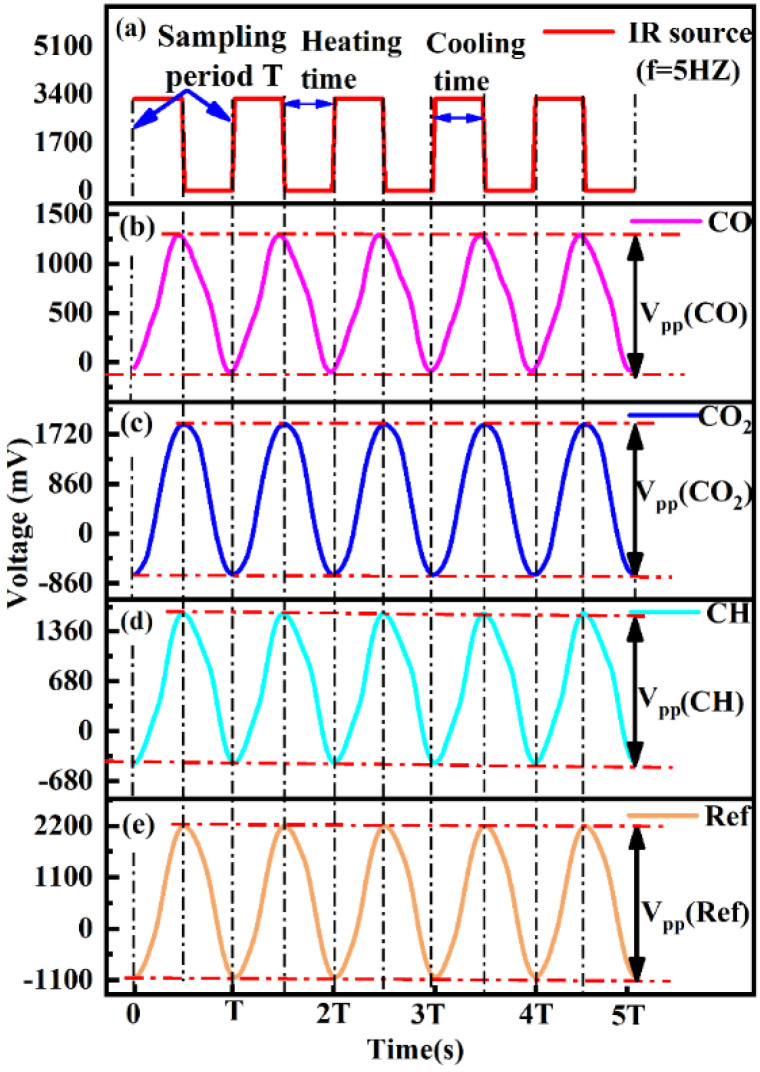
(**a**–**e**) Relationship between gas active channel, reference channel, and modulating pulse signals.

Figure 4a–e show the relationship between the modulating pulse signals, CO gas active channel, CO_2_ gas active channel, C_3_H_8_ gas active channel, and reference channel, respectively. In Figure 4a, the frequency and duty cycle were 5 Hz and 50%, respectively. The output of the modulation signal was altered, with a change in the operational state of the light source. Figure 4b–d are the output signals of the detector with the same frequency of modulation signal; the peak-to-peak amplitude of voltage (V_p-p_) was equal to the maximum value of the output signal minus the minimum value. In Figure S1, the V_p-p_ of the detector gradually decreased with an increase in the gas concentration. U_gas_ and U_ref_ are the peak-to-peak values of the target gas channel and the reference channel, respectively.

The detection range of concentration in this work and in other works are shown in Table 2.

## 4. Experimental Results and Discussion

### 4.1. Calibration and Data-Fitting

In this gas detection system, to obtain the concentration calculation model of each gas component, each gas channel should be calibrated separately. Standard gases of CO_2_, CO, and C_3_H_8_ at the concentrations of 20%, 14%, and 5000 ppm were prepared, respectively. The gas flow rate injected in the gas chamber was controlled at 700 mL/min by mass flow controllers (MFCs). The experiment was conducted at room temperature (20 ± 2 °C) and atmospheric pressure. The interference due to ambient temperature and pressure were negligible.

**Figure 5:**
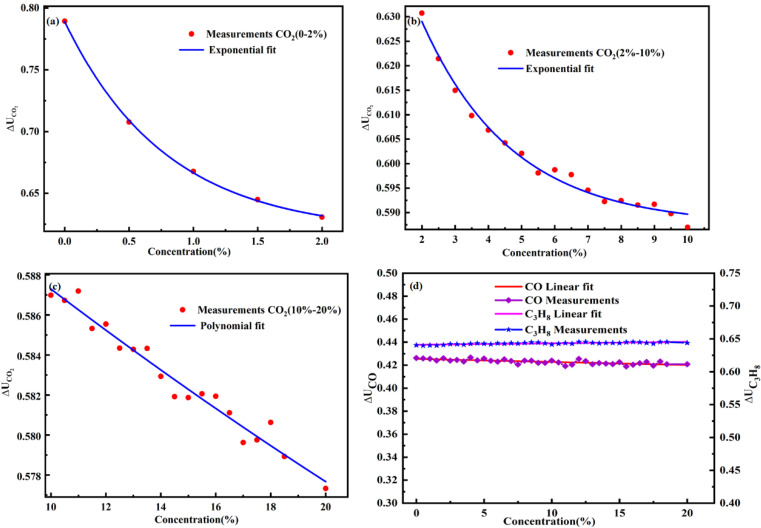
Experimental data of the relation between ΔU_gas_ and C and the piecewise fitting curve versus (**a**) CO_2_ (0–2%); (**b**) CO_2_ (2–10%); (**c**) CO_2_ (10–20%); (**d**) Cross interference of CO_2_ on CO and C_3_H_8_.

The experiment was conducted as follows:

The gas chamber was filled with pure (99.99%) nitrogen for 15 min. Subsequently, the standard gas was injected into the gas chamber at different concentrations, and the V_p-p_ and gas concentration were measured after 2 min. The concentration calculation model was trained using the least squares method. The model was validated using the standard gas, and the relative error of the experiment results was calculated. In this work, 39 different concentrations of CO_2_ were used to train the concentration calculation model. The detailed experimental data are shown in Table S1. Piecewise fitting was used to avoid overfitting or underfitting, thus improving the concentration calculation model. In the concentration ranges of 0–2%, 2–10%, and 10–20%, the experimental data and nonlinear fitting curve between ΔU_gas_ and C are shown in Figure 5a–c, and the piecewise fitting equations are Equations (9)–(11), respectively.

(9) $$\Delta U_{{\mathrm{CO}}_{2}}=0.17093\cdot \exp\left(-C/7947.626\right)+0.61801\;(R^{2}=99.97\%)$$

where R^2^ represents the quality of the curve fitting.

(10) $$\Delta U_{{\mathrm{CO}}_{2}}=0.087\cdot \exp\left(-C/27214.7\right)+0.5875\;(R^{2}=98.80\%)$$

(11) $$\Delta U_{{\mathrm{CO}}_{2}}=0.59849+(-0012)C+({7.937E-6)C}^{2}\;(R^{2}=96.39\%)$$

### 4.2. Cross Interference

Anti-cross sensitivity is an important indicator in a multi-gas analysis system. Additionally, the interference coefficient between different gases should be tested. Therefore, the interference models between these three gases were established using the least squares method. Considering CO_2_ as an example, CO_2_ with different concentrations was injected into the gas chamber. However, the concentration data of CO and C_3_H_8_ were also obtained because the four channels were simultaneously measured. The interference caused by CO_2_ on the CO and C_3_H_8_ channels can be determined by analyzing the measurement results of the CO and C_3_H_8_ channels.

Although the bandwidth of the filter was considerably narrow, the occurrence of cross interference between the three gas channels was inevitable. This study established the following model to describe the interference between the different gas channels—the concentration model of each channel is calculated by Equations (12)–(14), respectively:

(12) $$c\left({\mathrm{CO}}_{2}\right){=c}_{{\mathrm{CO}}_{2}}{+K}_{{\mathrm{CO}}_{2,}\mathrm{CO}\;}{+K}_{{\mathrm{CO}}_{2,}C_{3}H_{8}}$$

(13) $$c\left(\mathrm{CO}\right){=c}_{\mathrm{CO}}{+K}_{{\mathrm{CO},\mathrm{CO}}_{2}}{+K}_{{\mathrm{CO},C}_{3}H_{8}}$$

(14) $${c(C}_{3}H_{8}{)=c}_{C_{3}H_{8}}{+K}_{C_{3}H_{8}{,\mathrm{CO}}_{2\;}}{+K}_{C_{3}H_{8},\mathrm{CO}}$$

where c(CO_2_), c(CO), and c(C_3_H_8_) are the measured concentrations, and $c_{{\mathrm{CO}}_{2}}$, c_CO_, and $c_{C_{3}H_{8}}$ are the actual concentrations. $K_{{\mathrm{CO}}_{2,}\mathrm{CO}}$ is the effect coefficient of CO on CO_2_. To study the effect of CO_2_ on CO and C_3_H_8_, the experimental results and linear fitting curve are both shown in Figure 5d; the linear fitting curve equations between ΔU_gas_ and C are $\Delta U_{C_{3}H_{8}}$ Equation (15) and ΔU_CO_ Equation (16), respectively.

(15) $$\Delta U_{C_{3}H_{8}}=0.641\;-\;0.0002158\cdot c_{{\mathrm{CO}}_{2}}$$

(16) $$\Delta U_{\mathrm{CO}}=0.425+0.0002642\cdot c_{{\mathrm{CO}}_{2}}$$

In Figure 5d, as the CO_2_ concentration increases, the output value of the C_3_H_8_ channel and CO channel remain almost constant. This result indicates that CO_2_ had a minor effect on C_3_H_8_ and CO. Therefore, the effect coefficient of ${\mathrm{CO}}_{2}$ on the C_3_H_8_ and CO channel are $K_{C_{3}H_{8}{,\mathrm{CO}}_{2}}$ in Equation (17) and $K_{{\mathrm{CO},\mathrm{CO}}_{2}}$ in Equation (18), respectively.

(17) $$K_{C_{3}H_{8}{,\mathrm{CO}}_{2}}=(-0.0002158)\cdot c_{{\mathrm{CO}}_{2}}$$

(18) $$K_{{\mathrm{CO},\mathrm{CO}}_{2}}=0.0002642\cdot c_{{\mathrm{CO}}_{2}}$$

**Figure 6:**
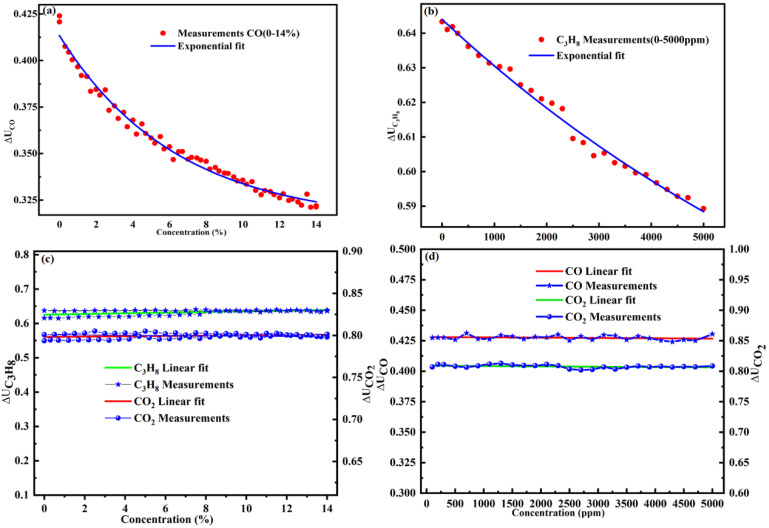
Measurements and fitting curve of the ratio ΔU_gas_ versus (**a**) CO; (**b**) C_3_H_8_; (**c**) Cross interference of CO on CO_2_ and C_3_H_8_; (**d**) Cross interference of C_3_H_8_ on CO_2_ and C_3_H_8_.

In total, 59 concentrations of CO were used to train the concentration calculation model. The detailed experimental data are shown in Table S3. For C_3_H_8_ sensing, a series of gas samples with different concentration levels (with an increment of 200 ppm) were prepared and passed into the gas chamber. For CO and C_3_H_8_ sensing, the obtained relationship curves between voltage ratio ΔU_gas_ and concentration C are shown in Figure 6a,b, respectively. The concentration inversion models of CO and C_3_H_8_ are given by Equations (19) and (20), respectively.

(19) $$\Delta U_{\mathrm{CO}}=0.1\cdot \exp\left({-c}_{\mathrm{CO}}/63576.3\right)+0.31\;(R^{2}=98.59\%)\;$$

(20) $$\Delta U_{C_{3}H_{8}}=0.14\cdot \exp\left({-c}_{C_{3}H_{8}}/9895.9\right)+0.50394\;(R^{2}=99\%)\;$$

To study the cross interference of the three gas channels, the experimental results of the relation between ΔU_gas_ and C, and the linear fitting curve are shown in Figure 6c,d. The linear fitting curve equations are Equations (21)–(24), respectively.

(21) $$\Delta U_{{\mathrm{CO}}_{2}/\mathrm{REF}}=0.798+0.0001602\cdot c_{\mathrm{CO}}$$

(22) $$\Delta U_{C_{3}H_{8}/\mathrm{REF}}=0.625+0.0009623\cdot c_{\mathrm{CO}}$$

(23) $$\Delta U_{{\mathrm{CO}}_{2}/\mathrm{REF}}=0.809+(-{5.153\;\times \;10}^{-7})\cdot c_{C_{3}H_{8}}$$

(24) $$\Delta U_{\mathrm{CO}/\mathrm{REF}}=0.428+(-{2.5067\;\times \;10}^{-7})\cdot c_{C_{3}H_{8}}$$

As shown in Figure 6c, as the gas concentration changes, the mutual interference also alters relatively, indicating that there is a mutual interference between the gas channels. It can be observed, from Figure 6d, that the detector output voltage ratio remains constant with an increase in the gas concentration. Therefore, the interference factors of gas channels are Equations (25)–(28), respectively.

(25) $$K_{{\mathrm{CO}}_{2,}\mathrm{CO}\;}=0.0001602\cdot c_{\mathrm{CO}}$$

(26) $$K_{C_{3}H_{8},CO}=0.0009623\cdot c_{\mathrm{CO}}$$

(27) $$K_{{\mathrm{CO}}_{2\;},C_{3}H_{8}}=(-{5.153\;\times \;10}^{-7})\cdot c_{C_{3}H_{8}}\;\approx \;0$$

(28) $$K_{{\mathrm{CO},\;C}_{3}H_{8}}=(-{2.5067\;\times \;10}^{-7})\;\approx \;0$$

Therefore, Figure 5d and Figure 6c,d indicate the presence of cross interference between C_3_H_8_, CO, and CO_2_. Hence, the interference models between these three gases are given by Equations (29)–(31), respectively.

(29) $$c\left({\mathrm{CO}}_{2}\right){=c}_{{\mathrm{CO}}_{2}}+0.0001602\cdot c_{\mathrm{CO}}$$

(30) $$c\left(\mathrm{CO}\right){=c}_{\mathrm{CO}}+0.0002642\cdot c_{{\mathrm{CO}}_{2}}$$

(31) $${c(C}_{3}H_{8}{)=c}_{C_{3}H_{8}\;}+(-0.0002158)\cdot c_{{\mathrm{CO}}_{2}}+0.0009623\cdot c_{\mathrm{CO}}$$

### 4.3. Relative Error

To further verify the accuracy of the data processor of the calibrated detector, seven sets of mixed gas measured results are shown in Table 3. The relative error (δ) was calculated using Equation (32).

(32) $${\delta =(C}_{m}{-\;C}_{s})/R\;\times 100\%$$

where C_m_ and C_s_ are measurement concentration and true concentration, respectively. R denotes the full scale.

Table 3 shows the CO_2_ concentration between 0.7% and 6%, and the measurement results smaller than true values; the deviation was between −0.15% and −0.55%. When the CO concentration was between 1 and 2%, the deviation was between −0.36% and −2.29%. When the C_3_H_8_ concentration increased from 500 ppm to 2000 ppm, the deviation decreased from 2.88% to 1.68%. The largest deviation was 3.06%, which was observed at a concentration of 2500 ppm, whereas the smallest deviation was 0.48%, which was observed at a concentration of 3000 ppm. According to the Beer–Lambert law, monochromatic radiation should ideally be used. However, it can be observed that owing to the limitation of the production technology, the infrared narrowband filter separated from the continuous radiation light emitted by the light source had a wavelength range with a certain bandwidth, not a single chromatographic line. Meanwhile, the transmittance of different types of filters in each channel is not ideal, and these factors will cause differences between the theoretical and actual measurements.

### 4.4. Interference Test

To further verify the accuracy of the interference models, the interfering gases (NO and NO_2_) concentrations were fixed at 100 ppm, the three target gases (C_3_H_8_, CO_2_, and CO) concentrations were changed, and so was the concentration ratio, as shown in Table 4.

In Table 4, when the interfering gases NO and NO_2_ are added, and C_3_H_8_ concentration was between 500 ppm and 2500 ppm, the measurement results of C_3_H_8_ from 638 ppm increased to 2598 ppm, and the deviation changed from 1.30% to 2.76%. However, when the CO_2_ concentration changed from 0.7% to 6%, the measurement results of CO_2_ increased from 0.65% to 5.54%, and the deviation was less than −2.40%. When CO concentration increased from 1% to 2%, the measurement results of CO increased from 0.79% to 1.84%, and the deviation was less than −1.60%. Table 4 indicates that when the interference gases NO and NO_2_ are added, the relative errors of the three target gases (C_3_H_8_, CO_2_ and CO) are all less than 2.77%. Table 4 proves that the interference models are successful.

### 4.5. Repeatability

To further verify the repeatability of the system, the measurements obtained three groups of data. Each group was performed 10 times, and the experimental data of continuous measurement C_3_H_8_, CO_2_, and CO are shown in Tables S4–S6. The standard deviation (σ) and relative standard deviation C_v_ was calculated using Equations (33) and (34), respectively.

(33) $$\sigma \;=\;\sqrt{\frac{\sum_{i=1}^{n}{\left(C_{i}-\bar{C}\right)}^{2}}{n-1}}$$

where C_i_ and $\bar{C}$ are the measurement concentration and average value of the measurement concentration, respectively.

(34) $$C_{V}=\sigma /C\;\times \;100\%$$

where C is the true concentration. The relative standard deviation C_v_ was used to evaluate the system’s repeatability.

As shown in Tables S3–S5, the mutual interference changes with a change in the gas concentration. As each channel of the detector has different photoelectric conversion effects for infrared light of different wavelengths and different half-widths, this caused differences in the detection accuracy of each channel. The average deviation of C_3_H_8_, CO_2_, and CO was expressed by relative true deviation, which was below 3.81%, 2.90%, and 4.5%, respectively. Therefore, this indicated that the NDIR system had modest properties.

### 4.6. Stability

A long-term stability detection of the multi-gas sensor was performed in a simulation environment inside the chamber. Initially, the chamber was cleaned by N_2_ for 15 min. Subsequently, the concentration of CO, CO_2_, and C_3_H_8_ in the chamber were set as 2%, 2%, and 2500 ppm, respectively. The sampling interval, i.e., the detection period, was set to 1 day, and 10 data points for each gas were collected in 10 days. The experimental data of continuous measurement CO_2_, CO, and C_3_H_8_ are shown in Table S6.

Based on Table S6, the stability δ_s_ for the multi-gas sensor can be calculated through Equation (35).

(35) $$\delta_{s}{=(C}_{\max}-C)/R\;\times \;100\%$$

where C and C_max_ are the true concentration and maximum drift concentration, respectively. R denotes the full scale.

The instability of the sensitivity of the pyroelectric detector leads to a difference in the accuracy of each sampling, and the sensitivity of the detector changes irregularly, which might lead to erroneous measurement results. It can be observed from Table S6 that the stability of the system was between −0.72 and −2.7%. Therefore, the system had a modest stability.

### 4.7. Response Time

The response time of the sensor refers to the time required for the output to stabilize after the input variable enters the sensor. In this experiment, first pass pure nitrogen was added into the gas chamber. After the sensor zero point was stable, we stopped passing the nitrogen and passed in the C_3_H_8_ with a concentration of 1000 ppm, the CO2 with a concentration of 2%, and the CO with a concentration of 1%. We recorded the display values from the beginning to the stable state. After measuring 5 times, the average response time was less than 11 s.

## 5. Conclusions

In summary, this study developed a three-gas detection system based on a single broadband light source and a four-channel pyroelectric detector using the principle of NDIR. The detection system was economical, and it simultaneously measured the concentrations of CO_2_, CO, and C_3_H_8_. The sensor calibration was studied, and the calibration method was verified. The interference models between these three gases were established using the least squares method. Finally, the experimental results demonstrated that the NDIR system exhibited good precision, modest stability, good repeatability, compactness, and long service life. It is power-efficient and a promising method for the analysis of automobile exhaust gases.

## Figures and Tables

**Table 1:** The characteristic parameters of the four-channel filter.

Target Gas	Absorption Peak (µm)	Central Wavelength (CWL) (µm)	Half-Power Bandwidth (HPB) (nm)
C_3_H_8_	3.39	3.33	160
CO	4.70	4.74	140
CO_2_	4.25	4.26	180
Ref gas	no	3.95	90

**Table 2:** The detection range of concentration compared with other works.

Method	TARGET GAS	The Range of Concentration	Reference
NDIR	CO_2_	0–4.8%	[20]
	CO	0–4.45%	
	CH_4_	0–5%	
NDIR	CO, CO_2_, CH_4_, H_2_CO, NH_3_, NO	0–4%	[24]
NDIR	CO, CO_2_, CH_4_	0–0.25%	[22]
NDIR	CO_2_	0–20%	This work
	CO	0–14%	
	C_3_H_8_	0–0.5%	

**Table 3:** Mixed gas test.

Gas Composition	True Concentration	Measurement Concentration	Deviation (%)
C_3_H_8_(ppm)	500	644	2.88
CO_2_(%)	1	0.89	−0.55
CO(%)	1	0.95	−0.36
C_3_H_8_(ppm)	980	1033	1.06
CO_2_(%)	6	5.69	−1.55
CO(%)	2	1.68	−2.29
C_3_H_8_(ppm)	1000	1098	1.96
CO_2_(%)	1.2	1.12	−0.40
CO(%)	1	0.90	−0.71
C_3_H_8_(ppm)	1500	1584	1.68
CO_2_(%)	1	0.95	−0.25
CO(%)	2	1.70	−2.14
C_3_H_8_(ppm)	2000	2084	1.68
CO_2_(%)	2	1.93	−0.35
CO(%)	2	1.81	−1.36
C_3_H_8_(ppm)	2500	2653	3.06
CO_2_(%)	0.70	0.67	−0.15
CO(%)	1	0.81	−1.36
C_3_H_8_(ppm)	3000	3024	0.48
CO_2_(%)	0.70	0.60	−0.50
CO(%)	2	1.89	−0.79

**Table 4:** Interference test.

C_3_H_8_(ppm)/NO(ppm)/NO_2_(ppm)	500/100/100	1000/100/100	1500/100/100	2000/100/100	2500/100/100
Measurements (C_3_H_8_)	638	1065	1567	2068	2598
Deviation (%)	2.76	1.30	1.34	1.36	1.96
CO_2_(%)/NO(ppm)/NO_2_(ppm)	0.7/100/100	1/100/100	1.2/100/100	2/100/100	6/100/100
Measurements (CO_2_)	0.65	0.98	1.18	1.65	5.54
Deviation (%)	−0.25	−0.10	−0.10	−1.75	−2.30
CO(%)/NO(ppm)/NO_2_(ppm)	1/100/100	2/100/100	1/100/100	2/100/100	1/100/100
Measurements (CO)	0.79	1.84	0.91	1.83	0.85
Deviation (%)	−1.50	−1.14	−0.64	−1.21	−1.07

## Data Availability

The study did not report any data.

## Supplementary Materials

The following supporting information can be downloaded at: https://www.mdpi.com/article/10.3390/s22030836/s1, Figure S1. Relationship between voltage and “sampling time. Table S1. CO_2_ experimental data. Table S2. CO experimental data. Table S3. Repeatability test I. Table S4. Repeatability test II. Table S5. Repeatability test III. Table S6. Stability test.
