# Supplementary material for: Multi-Gas Detection System Based on Non-Dispersive Infrared (NDIR) Spectral Technology
Source: Sensors (Basel). 2022 Jan 22;22(3):836. doi: 10.3390/s22030836 (PMC8838934; doi:10.3390/s22030836)
Supplement: Supplementary file 1 [file sensors-22-00836-s001.zip › sensors-1545571-supplementary.pdf]

sensors

MDPI

Supporting information

Multi-Gas Detection System Based on Non-Dispersive Infrared (NDIR) Spectral Technology

Manlin Xu [1], Bo Peng [2], Xiangyi Zhu [1] and Yongcai Guo [1,*]

[1] Key Laboratory of Optoelectronic Technology and Systems of Ministry of Education, College of Optoelectronic Engineering, Chongqing University, Chongqing 400044, China; manlinxu@cqu.edu.cn (M.X.); XiangyiZhu@cqu.edu.cn (X.Z.)

[2] Liangjiang School of Artificial Intelligence, Chongqing University of Technology, Chongqing 401135, China; pengbo86@126.com

* Correspondence: ycguo@cqu.edu.cn

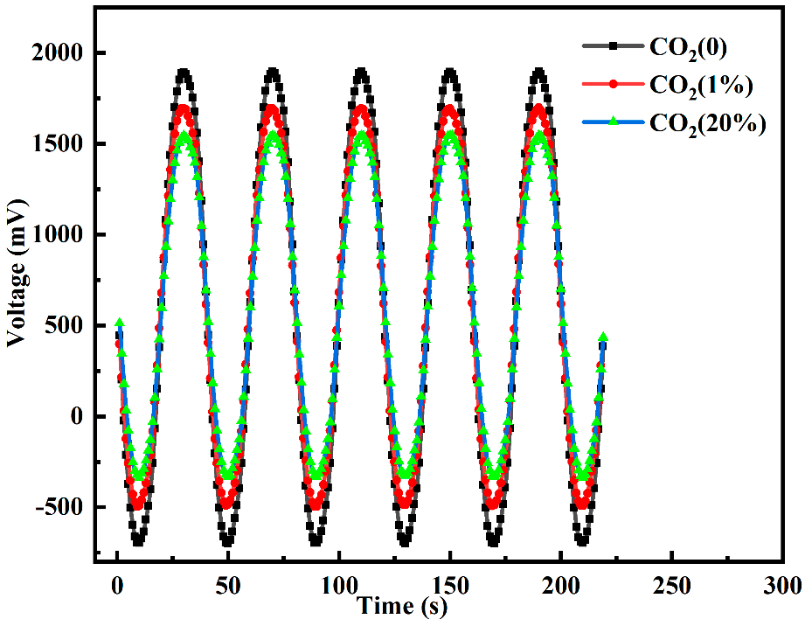

Figure S1. Relationship between voltage and sampling time.

Table S1. CO_2 experimental data.

Truth concentration (%)	Voltage ratio	Truth concentration (%)	Voltage ratio	Truth concentration (%)	Voltage ratio
0	0.78929	6.5	0.59776	13	0.58428
0.5	0.70779	7	0.59457	13.5	0.58433
1	0.66766	7.5	0.59225	14	0.58293
1.5	0.64489	8	0.59244	14.5	0.58192
2	0.63075	8.5	0.59154	15	0.58187
2.5	0.62145	9	0.59169	15.5	0.58206
3	0.61496	9.5	0.58979	16	0.58194
3.5	0.60983	10	0.58698	16.5	0.58111
4	0.60687	10.5	0.58672	17	0.57962
4.5	0.60422	11	0.58719	17.5	0.57975
5	0.60207	11.5	0.58532	18	0.58063
5.5	0.59809	12	0.58554	18.5	0.57893

6	0.59874	12.5	0.58434	20	0.57733

Table S2. CO experimental data.

Truth concentration (%)	Voltage ratio	Truth concentration (%)	Voltage ratio	Truth concentration (%)	Voltage ratio
0	0.42071	9.7	0.33531	5	0.35832
0.3	0.40761	10.2	0.33353	5.5	0.35913
0.7	0.40038	10.7	0.33023	6	0.35365
1.2	0.3919	11.2	0.33012	6.5	0.35103
1.7	0.38343	11.7	0.32792	7	0.34695
2.2	0.38137	12.2	0.32837	7.5	0.34761
2.7	0.37327	12.7	0.32553	8	0.34581
3.2	0.36888	13.2	0.32229	8.5	0.34256
3.7	0.36442	13.7	0.32118	9	0.33952
4.2	0.36046	14	0.32129	9.5	0.33748
4.7	0.36083	0	0.42395	10	0.33577
5.2	0.35555	0.5	0.40448	10.5	0.33492
5.7	0.35251	1	0.39661	11	0.32785
6.2	0.34679	1.5	0.39143	11.5	0.32956
6.7	0.35111	2	0.38446	12	0.32624
7.2	0.34794	2.5	0.38422	12.5	0.32482
7.7	0.34647	3	0.3756	13	0.32405
8.2	0.3417	3.5	0.37221	13.5	0.32819
8.7	0.34062	4	0.36798	14	0.32196
9.2	0.3393	4.5	0.36593		

Table S3. Repeatability test Ⅰ.

Gas	True concentration	Measurement concentration					Average value	Average deviation (%)
**C3H8 (ppm)**	**2000**	**2016**	**2084**	1986	1966	2084	2023	3.39
		1985	2169	2014	1976	1958		
CO2(%)	2	1.93	1.99	1.88	1.86	1.98	1.91	2.90
		1.88	1.93	1.94	1.82	1.84		
CO(%)	2	1.83	1.78	1.92	1.76	1.86	1.79	4.50
		1.80	1.68	1.86	1.62	1.74		

Table S4. Repeatability test Ⅱ.

Gas	True concentration	Measurement concentration					Average value	Average deviation (%)
**CH(ppm)**	**2500**	**2599**	**2380**	2472	2540	2556	2538	3.81
		2585	2391	2674	2600	2586		
CO2(%)	1.2	1.09	1.09	1.11	1.11	1.09	1.09	1.04
		1.10	1.07	1.09	1.09	1.10		
CO(%)	1	0.81	0.87	0.80	0.85	0.80	0.84	4.44
		0.86	0.90	0.91	0.85	0.78		

Table S5. Repeatability test III.

Gas	True concentration	Measurement concentration					Average value	Average deviation (%)
**C_3H_8(ppm)**	**3500**	**3565**	**3454**	3409	3509	3417	3486	1.83
		3590	3485	3408	3519	3506		
CO_2(%)	0.7	0.59	0.58	0.58	0.58	0.58	0.58	1.26
		0.58	0.59	0.56	0.57	0.58		
CO(%)	2	1.62	1.75	1.87	1.69	1.79	1.77	4.37

Table S6. Stability test.

**Gas**	**Measurement concentration**										**Average value**	**Stability (%)**
	**1day**	**2day**	**3day**	**4day**	**5day**	**6day**	**7day**	**8day**	**9day**	**10day**		
C3H8(ppm)	2560	2526	2595	2521	2520	2515	2513	2533	2395	2646	2532	-2.11
CO2(%)	1.92	1.87	1.93	1.91	1.95	1.86	1.97	1.96	1.92	2.01	1.93	-0.72
CO(%)	1.87	1.75	2.04	1.66	1.63	1.67	2.05	1.93	1.97	1.77	1.83	-2.63
